# Correction to: Fetal Membrane Inflammation Induces Preterm Birth Via Toll-Like Receptor 2 in Mice With Chronic Gingivitis

**DOI:** 10.1007/s43032-021-00706-z

**Published:** 2021-09-14

**Authors:** Haruhisa Konishi, Satoshi Urabe, Hiroshi Miyoshi, Yuko Teraoka, Tomoko Maki, Hisako Furusho, Mutsumi Miyauchi, Takashi Takata, Yoshiki Kudo, Shunichi Kajioka

**Affiliations:** 1 Department of Obstetrics and Gynecology, Graduate School of Biomedical Sciences and Health Sciences, Hiroshima University, Hiroshima, Japan; 2 Department of Obstetrics and Gynecology, Hiroshima Prefectural Hospital, Hiroshima, Japan; 3 Department of Urology, Graduate School of Medical Sciences, Kyushu University, Fukuoka, Japan; 4 Department of Applied Urology and Molecular Medicine, Graduate School of Medical Sciences, Kyushu University, Fukuoka, Japan; 5 Department of Oral and Maxillofacial Pathobiology, Basic Life Sciences, Graduate School of Biomedical and Health Sciences, Hiroshima University, Hiroshima, Japan

**Correction to: Reproductive Sciences**

**https://doi.org/10.1177/1933719118792097**

The article “Fetal Membrane Inflammation Induces Preterm Birth Via Toll-Like Receptor 2 in Mice With Chronic Gingivitis,” written by Haruhisa Konishi, Satoshi Urabe, Hiroshi Miyoshi, Yuko Teraoka, Tomoko Maki, Hisako Furusho, Mutsumi Miyauchi, Takashi Takata, Yoshiki Kudo, and Shunichi Kajioka, was originally published Online First without Open Access. After publication in volume 26, issue 7, pages 869–878, the authors decided to opt for Open Choice and to make the article an Open Access publication. Therefore, the copyright of the article has been changed to © The Author(s) 2021 and the article is forthwith distributed under the terms of the Creative Commons Attribution 4.0 International License, which permits use, sharing, adaptation, distribution and reproduction in any medium or format, as long as you give appropriate credit to the original author(s) and the source, provide a link to the Creative Commons licence, and indicate if changes were made. The images or other third party material in this article are included in the article’s Creative Commons licence, unless indicated otherwise in a credit line to the material. If material is not included in the article’s Creative Commons licence and your intended use is not permitted by statutory regulation or exceeds the permitted use, you will need to obtain permission directly from the copyright holder. To view a copy of this licence, visit http://creativecommons.org/licenses/by/4.0

